# A rare large duplication of *MLH1* identified in Lynch syndrome

**DOI:** 10.1186/s13053-021-00167-0

**Published:** 2021-01-19

**Authors:** Abhishek Kumar, Nagarajan Paramasivam, Obul Reddy Bandapalli, Matthias Schlesner, Tianhui Chen, Rolf Sijmons, Dagmara Dymerska, Katarzyna Golebiewska, Magdalena Kuswik, Jan Lubinski, Kari Hemminki, Asta Försti

**Affiliations:** 1 Division of Molecular Genetic Epidemiology, German Cancer Research Center (DKFZ), Im Neuenheimer Feld 580, D-69120 Heidelberg, Germany; 2 Institute of Bioinformatics, International Technology Park, Bangalore, 560066 India; 3 Manipal Academy of Higher Education (MAHE), Manipal, Karnataka 576104 India; 4 Computational Oncology, Molecular Diagnostics Program, National Center for Tumor Diseases (NCT), Heidelberg, Germany; 5 Hopp Children’s Cancer Center (KiTZ), Heidelberg, Germany; 6 Division of Pediatric Neurooncology, German Cancer Research Center (DKFZ), German Cancer Consortium (DKTK), Im Neuenheimer Feld 580, 69120 Heidelberg, Germany; 7 Bioinformatics and Omics Data Analytics, German Cancer Research Center (DKFZ), Heidelberg, Germany; 8 Department of Cancer Prevention, Cancer Hospital of the University of Chinese Academy of Sciences (Zhejiang Cancer Hospital), Institute of Cancer and Basic Medicine, Chinese Academy of Sciences, Hangzhou, China; 9 Department of Genetics, University Medical Center Groningen, University of Groningen, Groningen, The Netherlands; 10 Hereditary Cancer Center, Department of Genetics and Pathology, Pomeranian Medical University, 70-111 Szczecin, Poland; 11 Faculty of Medicine and Biomedical Center in Pilsen, Charles University in Prague, 30605 Pilsen, Czech Republic; 12 Division of Cancer Epidemiology, German Cancer Research Center (DKFZ), Heidelberg, Germany

**Keywords:** Genetic predisposition, Lynch syndrome, Mismatch repair genes, Whole-genome sequencing

## Abstract

**Background:**

The most frequently identified strong cancer predisposition mutations for colorectal cancer (CRC) are those in the mismatch repair (MMR) genes in Lynch syndrome. Laboratory diagnostics include testing tumors for immunohistochemical staining (IHC) of the Lynch syndrome-associated DNA MMR proteins and/or for microsatellite instability (MSI) followed by sequencing or other techniques, such as denaturing high performance liquid chromatography (DHPLC), to identify the mutation.

**Methods:**

In an ongoing project focusing on finding Mendelian cancer syndromes we applied whole-exome/whole-genome sequencing (WES/WGS) to 19 CRC families.

**Results:**

Three families were identified with a pathogenic/likely pathogenic germline variant in a MMR gene that had previously tested negative in DHPLC gene variant screening. All families had a history of CRC in several family members across multiple generations. Tumor analysis showed loss of the MMR protein IHC staining corresponding to the mutated genes, as well as MSI. In family A, a structural variant, a duplication of exons 4 to 13, was identified in *MLH1*. The duplication was predicted to lead to a frameshift at amino acid 520 and a premature stop codon at amino acid 539. In family B, a 1 base pair deletion was found in *MLH1,* resulting in a frameshift and a stop codon at amino acid 491. In family C, we identified a splice site variant in *MSH2*, which was predicted to lead loss of a splice donor site.

**Conclusions:**

We identified altogether three pathogenic/likely pathogenic variants in the MMR genes in three of the 19 sequenced families. The *MLH1* variants, a duplication of exons 4 to 13 and a frameshift variant, were novel, based on the InSiGHT and ClinVar databases; the *MSH2* splice site variant was reported by a single submitter in ClinVar. As a variant class, duplications have rarely been reported in the MMR gene literature, particularly those covering several exons.

**Supplementary Information:**

The online version contains supplementary material available at 10.1186/s13053-021-00167-0.

## Background

Familial cancer, here defined as two or more first-degree relatives diagnosed with the same cancer, accounts for some 15% of colorectal cancer (CRC) [1]. The most frequently identified strong cancer predisposition mutations for CRC are those in mismatch repair (MMR) genes in Lynch syndrome, which account for approximately 1% of CRCs in the population (depending on the population) [2]. A number of other high-risk genes are known but variants in these are very rare [3]. In addition, ever-increasing numbers (> 100) of low-risk gene variants have been described for CRC [4]; yet combined, the high and low-risk variants explain only a small proportion of the known familial risk and even less of the heritability estimated in twin studies [5, 6].

Clinical diagnostics of Lynch syndrome usually first considers family history based on the Amsterdam and Bethesda criteria [7]. These are not perfect as half of germline-confirmed Lynch syndrome patients fail to meet the Amsterdam II criteria and, although the Bethesda guidelines are sensitive, their specificity is low [7]. Diagnostic laboratory tests include testing tumors for immunohistochemical (IHC) staining of the Lynch syndrome-associated DNA MMR proteins and/or for microsatellite instability (MSI) [7]. While these tests alone have a sensitivity ranging from 55 to 90% of predicting Lynch syndrome, combining the two will reach a sensitivity over 90% [7]. The identification of mutations is done by sequencing, or by other techniques, such as denaturing high performance liquid chromatography (DHPLC) or multiplex ligation dependent probe amplification (MLPA) for structural variants [8]. More recently, next generation sequencing panels have become the golden standard in identification of pathogenic germline variants in hereditary cancer syndromes. In a recent study, a universal 83-gene next generation sequencing panel identified nearly double as many pathogenic germline variants related to hereditary cancer syndromes as the guideline-directed targeted testing in unselected cancer patients, leading to a treatment change for nearly 30% of these patients [9]. This highlights the usefulness of next generation sequencing in the clinical praxis and compensates the limitations of the clinical and guideline-based risk assessment.

We have been involved in a whole-exome/whole-genome sequencing (WES/WGS) project aimed at identifying Mendelian type cancer syndromes in families referred to the Hereditary Cancer Center, Szczecin. In three families fulfilling the Amsterdam II criteria of Lynch syndrome with negative results in DHPLC mutation screening of the Lynch syndrome-related MMR genes we identified a mutation in these genes using whole genome sequencing. Here, we report these variants, particularly a large duplication in the *MLH1* gene, as these types of large structural variants, particularly insertions are rarely described in Lynch syndrome [10–14].

## Patients and methods

In several regions of Poland, population screening was performed mainly in years 2000–2014, in which questionnaires on cancer family history were collected systematically. Individuals with a positive CRC family history were invited to genetic outpatient clinics all over Poland and their more detailed family histories were taken through detailed face-to-face interviews. Nineteen families with strong CRC aggregation compatible with an autosomal dominant pattern of inheritance were recruited to the study. Each family had at least three pathologically confirmed CRC cases; 17 families had at least one case diagnosed below the age of 55 years. All 19 families had undergone DHPLC analysis for MMR variants with negative test results [15]. The ethical approval for this study design was obtained from the Bioethics Committee of the Pomeranian Medical Academy in Szczecin No: BN-001/174/05. Sample collection was performed following the guidelines proposed by this Committee. A written informed consent was signed by each participant in accordance with the Helsinki declaration.

WES on CRC patients and healthy family members of 5 families and WGS on 14 families was performed in the Illumina X10 platform using DNA extracted from the blood samples. WGS was carried out as paired-end sequencing with a read length of 150 bp. Sequences were mapped to the reference human genome (build hg19, assembly hs37d5) using BWA mem (version 0.7.8) and duplicates were marked using Picard (version 1.125). Single nucleotide variants and small indels were called by using Platypus (version 0.8.1) and annotated using ANNOVAR [16], dbSNP [17], 1000 Genomes phase III [18], dbNSFP v.2.9 [19], and ExAC [20], respectively. Variant filtering was carried out by considering a minimum of 5 reads coverage and a minimum QUAL score of 20. To check for family relatedness, a pairwise comparison of variants among the cohort was performed.

GATK gCNV module (version 4.1.7.0) was used to call germline copy number variants (gCNVs) from the WES/WGS samples individually against a background of 200 WGS samples sequenced from the sample platform. The gCNVs were called based on the best practice recommended by the GATK (https://gatk.broadinstitute.org/hc/en-us/articles/360035531152%2D%2DHow-to-Call-common-and-rare-germline-copy-number-variants). The major deviation from the above best practice was that the gCNVs cohort models were created only for the Gencode v19 exonic regions of WGS data by considering them as the target regions. The sequences of the samples from the CRC families were compared against this model. This decreased the turnaround time for the analysis of gCNVs from the WGS data.

The resulting gCNV segments with QS score above 30 were selected and annotated with the subset of gnomAD structural variant (SV) data (version 2.1, variants with ‘PASS’ filter tags and ‘DUP’ or ‘DEL’ SV types) using vcfanno [21]. The segments with at least 80% overlap with a common gnomAD SV (popmax MAF > 0.1%) of same SV subtype were considered as common and removed. In addition, to consider a gCNV as rare, at least 50% of the targets (exons here) in the gCNV segments should have the denoised ploidies among the bottom (in the case of deletion) or top (in the case of duplication) 5% of denoised cohort ploidies from the background cohort samples. Subsequently, the candidate rare gCNVs were selected if they followed the disease inheritance pattern in the family. For the candidate gCNVs the genomic breakpoints were manually reviewed using the Integrative Genomic Viewer (IGV) [22] to determine the genomic coordinates of the gCNVs.

Sequencing data were visually inspected using IGV to exclude false positive variants. For variants causing a frameshift, we used the Translate tool (https://web.expasy.org/translate/) to translate the nucleotide sequence to a protein sequence. The effect of splice site variants on splicing was analyzed using NetGene2 (http://www.cbs.dtu.dk/services/NetGene2/). Combined Annotation-Dependent Depletion (CADD) score was used to evaluate the deleteriousness of the variants; the scores > 20 and > 30 are indicative of the top 1% and top 0.1% of deleterious variants, respectively [23]. The InSiGHT database available at the Leiden Open Variation Database (LOVD) v.3.0 [24, 25], ClinVar (https://www.ncbi.nlm.nih.gov/clinvar/) [26], gnomAD database (https://gnomad.broadinstitute.org/) and the recent publication on Chinese MMR variants were used as a reference [11].

IHC and MSI analyses were performed as reported previously [8, 27] in CRC samples from individuals with a MMR gene variant detected through WES or WGS.

## Results

**Fig. 1:**
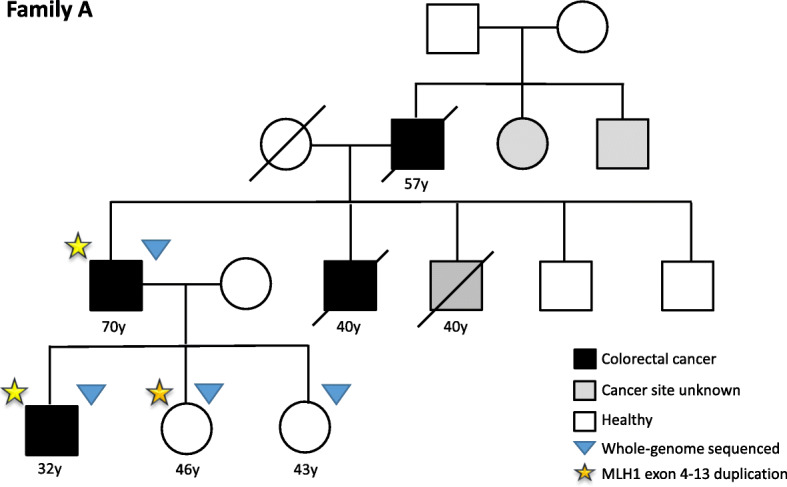
Pedigree of the colorectal cancer family with MLH1 exon 4–13 duplication

In three of the 19 families sequenced, pathogenic/likely pathogenic MMR gene variants were identified. The pedigree of family A is shown in Fig. 1. Several patients diagnosed with CRC were present in three generations. We sequenced the affected father (diagnosed at age 70 years) and his son (diagnosed at age 32 years). Additionally, two unaffected individuals were sequenced. The pedigrees of families B and C are found in **Additional file** **1****:** Fig. 1.

The detected variants are listed in Table 1. In family A, a structural variant, a duplication of chr3:37045366–37,071,869 covering exons 4 to 13 of *MLH1* was identified. It was predicted to lead to a frameshift at amino acid 520 and a premature stop codon at amino acid 539. The duplication was identified in both patients and in an unaffected female relative who was 9 years older than her affected brother. In family B, a one base pair deletion was found in *MLH1* which resulted in a frameshift and a stop codon at amino acid 491. In family C, the three affected individuals carried a splice site variant in *MSH2,* with a CADD score of 23.4 (Table 1). According to NetGene2, the *MSH2* variant c.792 + 1G > C lead to a loss of a splice donor site.

**Table 1:** Mismatch repair gene variants in three colorectal cancer families

Family	Gene	CHROM_POS_REF_ALT^a^	HGVS nomenclature^b^	ANNOVAR annotation	Protein change
Family A	*MLH1*	3_37,045,366–37,071,869_dup	LRG_216t1_216:c.307-526_1558 + 1446dup	duplication exons 4–13	p.(Val520Glyfs*19)
Family B	*MLH1*	3_37067362_AG_A	LRG_216t1:c.1274del	frameshift	p.(Arg425Serfs*66)
Family C	*MSH2*	2_47639700_G_C	LRG_218t1:c.792 + 1G > C^c^	splicing	

^a^ Human genome build hg19, assembly hs37d5

^b^ according to den Dunnen JT: HGVS Recommendations for the Description of Sequence Variants: 2016 Update, Hum Mutat 37:564–569, 2016

^c^ ClinVar c.792 + 1G > C, likely pathogenic, review status: criteria provided, single submitter (accession number VCV000951452.1)

**Fig. 2:**
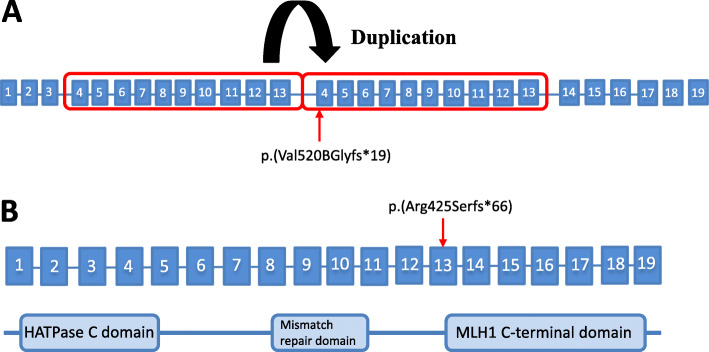
Graphic representation of the MLH1 structure describing the consequences and location of the MLH1 duplication and the frameshift variant. (a) MLH1 duplication of exons 4–13 in family A leads to a frameshift at amino acid 520 and a premature stop codon at amino acid 539. (b) MLH1 frameshift variant at amino acid 425 in family B leads to a premature stop codon at amino acid 491. Both variants lead to the deletion of the MLH1 C-terminal domain, which is needed for the MLH1-PMS2 heterodimerization

The IHC and MSI results of the tumor samples from the Lynch syndrome patients are shown in Table 2. Tumor samples from patients from families A and B did not express MLH1 and PMS2 proteins while in family C the tumor sample was negative for MSH2 and MSH6 proteins. The results are in line with the mutation analysis as MLH1 and PMS2 as well as MSH2 and MSH6 form heterodimers. Further in line, the MSI analysis showed MSI-high for families A and C; the analysis for family B failed. Capillary electrophoresis MSI diagrams for samples from families A and C are shown with pattern shifts for the monomorphic markers (**Additional file** **1****:** Fig. 2). The identical migration of the pentanucleotide markers confirms the sample identity.

**Table 2:** Immunohistochemistry (IHC) and microsatellite instability (MSI) analysis on tumor samples of the colorectal cancer families

Family	Variant	IHC MLH1	IHC PMS2	IHC MSH2	IHC MSH6	MSI
Family A	MLH1 duplication exons 4–13	negative	negative	positive	positive	high
Family B	MLH1 c.1274delG;p.Arg425Serfs*66	negative	negative	positive	positive	failed
Family C	MSH2 c.792 + 1G > C	positive	positive	negative	negative	high

The consequences of the *MLH1* variants on the gene and protein structure are shown in Fig. 2. In family A, the large duplication of exons 4 to13 covered a small section of the ATP binding domain (HATPase C domain) and the entire mismatch repair domain (MutL, i.e., MSH2-MLH1 heterodimer binding domain) as well as a small part of the MLH1 C-terminal domain (Fig. 2a). The duplication was predicted to lead to a frameshift at amino acid 520 and a premature stop codon at amino acid 539. Figure 2b shows the *MLH1* frameshift variant at amino acid 425 in family B leading to a premature stop codon at amino acid 491. Both variants were predicted to lead to the deletion of the MLH1 C-terminal domain, which is needed for the MLH1-PMS2 heterodimerization.

## Discussion

The present sequencing effort in families with a CRC family history suggestive of autosomal dominant inheritance identified two families with a pathogenic variant in the *MLH1* gene and one family with a likely pathogenic variant in the *MSH2* gene. In Poland, over 100 MMR gene point mutations have been identified, most of which are either frameshift or nonsense mutations leading to a truncated protein [28]. In over 60% of all Polish Lynch syndrome families a recurrent mutation is present. Two of the most frequent alterations are a substitution of A to T at the splice donor site of intron 5 of *MSH2* and a missense change (A681T) of *MLH1* [8]. In Polish patients, large deletions have been described particularly in the *MSH2* gene [8].

The present three variants have so far not been reported in InSiGHT [24, 25]; only the *MSH2* variant has been reported once in ClinVar [26]. However, the InSiGHT database lists similar *MLH1* variants causing a frameshift and leading to a protein truncation at approximately the same position as our variants, both have a classification “pathogenic” [24, 25] (Table 3). The *MSH2* variant is reported in ClinVar by a single submitter (accession number VCV000951452.1) and predicted to be “likely pathogenic” [26]. InSiGHT reports another nucleotide change at the same position, a c.792 + 1G > A variant, with a classification “pathogenic” (Table 3).

**Table 3:** Examples of InSiGHT DNA and protein changes for variants causing similar DNA and/or protein changes as the variants identified in the Polish families

Gene	CHROM_POS_REF_ALT^a^	InSiGHT DNA change [protein change]	InSiGHT class
MLH1	3_37070422-37070423_G_GT	c.1557_1558insT [p.(Val520Cysfs*8)]^b^	pathogenic
MLH1	3_37067349_TA_T	c.1261del [p.(Ser421Valfs*70)]^c^	pathogenic
MSH2	2_47639700_G_A	c.792 + 1G > A^d^	pathogenic

^a^ Human genome build hg19, assembly hs37d5

^b^ nearby position with similar consequence as caused by the large duplication in Family A

^c^ nearby position with similar consequence as caused by the frameshift variant in Family B

^d^ same position as the splice site variant in Family C, different nucleotide change

The present *MLH1* variants were predicted to cause protein truncation and to be pathogenic, while the *MSH2* splice site variant was predicted to be likely pathogenic. They add to the large collection of (likely) pathogenic variants in the MMR genes. Although all of these were unique, the duplication is of special interest as large duplications have rarely been reported for MMR genes. In the European literature somewhat over 10 exon level duplications have been reported, most of them in *MSH2* and fewer in *MLH1* [10, 12]. Similarly, in the recent Chinese literature survey on 34,000 individuals including both cancer cases and individuals without cancer, 540 MMR variants were found, but only 3 single exon duplications were reported for *MLH1* and one for *MSH2* [11]. In one of these papers the breakpoints implicated Alu mediated recombination as a mechanism and the duplication was predicted to create a premature stop codon and the formation of a truncated protein [12]. In the InSiGHT database, only 9 exon-level duplications in *MLH1* are reported compared to 77 deletions; in *MSH2* the numbers are 7 duplications and 84 deletions [24, 25] (**Additional file** **1****:** Table 1). While all the deletions had clinical classification “pathogenic”, only 2 duplications in *MLH1* and 3 in *MSH2* were classified as “pathogenic”. While large deletions most likely lead to non-functional proteins, the effect of large duplications may depend on whether the duplication is in-frame or not. The duplication in *MLH1* we present here is predicted to cause a frameshift and a truncated protein.

The present duplication of exons 4 through 13 covered a small section of the ATP binding domain (HATPase C domain), the entire mismatch repair domain (MutL, i.e., MSH2-MLH1 heterodimer binding domain) and part of the MLH1 C-terminal domain [29–31]. The out-of-frame change at amino acid 520 was predicted to cause a stop codon further down-stream at amino acid 539. Thus, the resulting truncated protein is probably degraded by non-sense mediated decay as supported by the IHC results of lack of MLH1 protein in the tumor. The C-terminal end of MLH1 contains important binding sites for heterodimeric MMR proteins that contribute to the various key functions such as endonuclease activity [30, 31].

The fact that these three mutations were missed in the previous screening early 2000s may be due to the methodology used at that time, DHPLC. The DHPLC primers were designed to cover all exons and approximately 30–60 bp upstream and downstream of each exon. As the breakpoints of the large duplication in *MLH1*were located 526 bp downstream of exon 4 and 1446 bp upstream of exon 13, it was missed. Also the splice site variant in *MSH2* may have been missed, because its distance to the upstream primer for detecting exon 4 was only 2 bp. Only the frameshift variant in *MLH1* was located in the middle of exon 12 and might have been possible to detect. This calls for the recommendation that historically negative cases, assessed by inferior methods, should be re-considered for testing using up-to-date methodologies.

## Conclusions

We identified three novel MMR gene variants that were predicted to lead to truncated proteins. The variants segregated with the disease and are expected to predispose to Lynch syndrome phenotypes, including CRC.

## Supplementary Information

**Additional file 1: Fig. S1.** (Family B) Pedigree of the colorectal cancer family with MLH1 frameshift variant. (Family C) Pedigree of the colorectal cancer family with MSH2 splice site variant. **Fig. S2.** Microsatellite instability (MSI) analysis of the tumor samples of two family members from Family A and one tumor sample from Family C. For each family, individuals with the tumor samples analyzed are indicated by an arrow and the MSI plots are shown for the corresponding germline and tumor samples. **Table S1.** Number of large deletions and duplications in the mismatch repair genes reported in the InSiGHT database and their clinical classification according to Mismatch Repair Gene Variant Classification Criteria by the InSiGHT Variant Interpretation Committee.

## Data Availability

The datasets generated and/or analysed during the current study are available from the corresponding author on reasonable request.

## Abbreviations

CRC: colorectal cancer
MMR: mismatch repair
IHC: immunohistochemical
MSI: microsatellite instability
DHPLC: denaturing high performance liquid chromatography
MLPA: multiplex ligation dependent probe amplification
WES/WGS: whole-exome/whole-genome sequencing
gCNV: germline copy number variant
SV: structural variant
IGV: Integrative Genomic Viewer
CADD: Combined Annotation-Dependent Depletion
