# Supplementary material for: A rare large duplication of MLH1 identified in Lynch syndrome
Source: Hered Cancer Clin Pract. 2021 Jan 19;19:10. doi: 10.1186/s13053-021-00167-0 (PMC7814444; doi:10.1186/s13053-021-00167-0)
Supplement: Supplementary file 1 — Additional file 1: Fig. S1. (Family B) Pedigree of the colorectal cancer family with MLH1 frameshift variant. (Family C) Pedigree of the colorectal cancer family with MSH2 splice site variant. Fig. S2. Microsatellite instability (MSI) analysis of the tumor samples of two family members from Family A and one tumor sample from Family C. For each family, individuals with the tumor samples analyzed are indicated by an arrow and the MSI plots are shown for the corresponding germline and tumor samples. Table S1. Number of large deletions and duplications in the mismatch repair genes reported in the InSiGHT database and their clinical classification according to Mismatch Repair Gene Variant Classification Criteria by the InSiGHT Variant Interpretation Committee. [file 13053_2021_167_MOESM1_ESM.pdf]

Additional file

A rare large duplication of *MLH1* identified in Lynch syndrome

Abhishek Kumar[1,2,3,#] , Nagarajan Paramasivam[4,#] , Obul Reddy Bandapalli[1,5,6], Matthias Schlesner[7], Tianhui Chen[8], Rolf Sijmons[9], Dagmara Dymerska[10], Katarzyna Golebiewska[10], Magdalena Kuswik[10], Jan Lubinski[10], Kari Hemminki[1,11,12,§] and Asta Försti[1,5,6,§,*]

[1] Division of Molecular Genetic Epidemiology, German Cancer Research Center (DKFZ), Im Neuenheimer Feld 580, D-69120, Heidelberg, Germany

[2] Institute of Bioinformatics, International Technology Park, Bangalore, 560066 India

[3] Manipal Academy of Higher Education (MAHE), Manipal, 576104, Karnataka, India

[4] Computational Oncology, Molecular Diagnostics Program, National Center for Tumor Diseases (NCT), Germany

[5] Hopp Children's Cancer Center (KiTZ), Heidelberg, Germany

[6] Division of Pediatric Neurooncology, German Cancer Research Center (DKFZ), German Cancer Consortium (DKTK), Heidelberg, Germany

[7] Bioinformatics and Omics Data Analytics, German Cancer Research Center (DKFZ), Heidelberg, Germany

[8] Department of Cancer Prevention, Cancer Hospital of the University of Chinese Academy of Sciences (Zhejiang Cancer Hospital), Institute of Cancer and Basic Medicine, Chinese Academy of Sciences, Hangzhou, China

[9] Department of Genetics, University Medical Center Groningen, University of Groningen, The Netherlands

[10] Hereditary Cancer Center, Department of Genetics and Pathology, Pomeranian Medical University, 70-111 Szczecin, Poland

[11] Faculty of Medicine and Biomedical Center in Pilsen, Charles University in Prague, 30605 Pilsen, Czech Republic

[12] Division of Cancer Epidemiology, German Cancer Research Center (DKFZ), Heidelberg, Germany

Additional file: Table 1 Number of large deletions and duplications in the mismatch repair genes reported in the InSiGHT database and their clinical classification according to Mismatch Repair Gene Variant Classification Criteria by the InSiGHT Variant Interpretation Committee.

Gene	Total number of deletions	Number of exon-level deletions	Clinical classification	Total number of duplications	Number of exon-level duplications	Clinical classification
MLH1	290	77	All pathogenic	80	9	2 pathogenic, 7 VUS
MSH2	276	84	All pathogenic	85	7	3 pathogenic, 4 VUS
MSH6	152	13	9 pathogenic, 4 VUS	77	1	VUS
PMS2	35	19	All pathogenic	6	0	

VUS, variant of uncertain significance

Additional file: Fig. 1 (Family B) Pedigree of the colorectal cancer family with MLH1 frameshift variant; (Family C) Pedigree of the colorectal cancer family with MSH2 splice site variant.

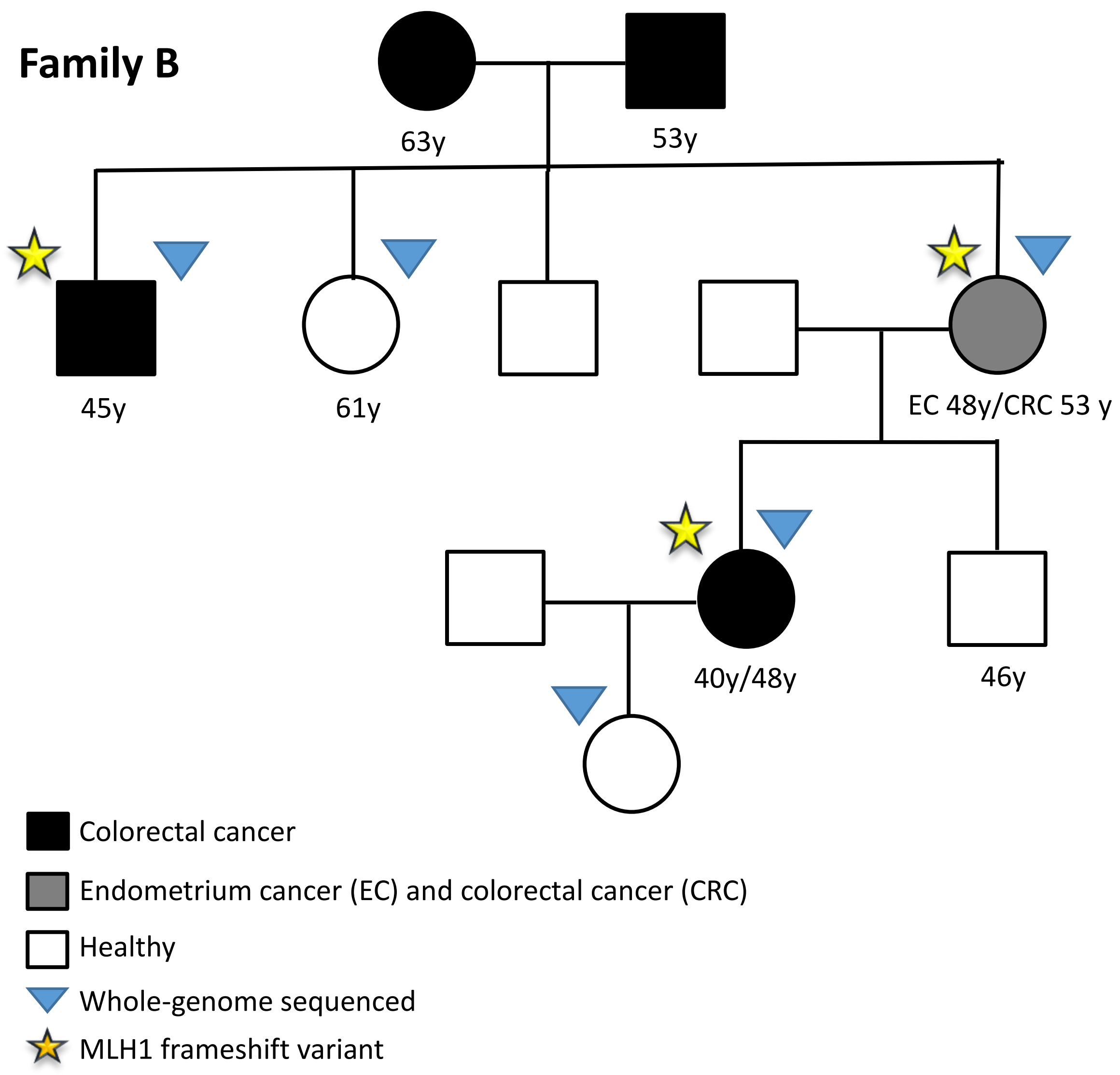

Family C

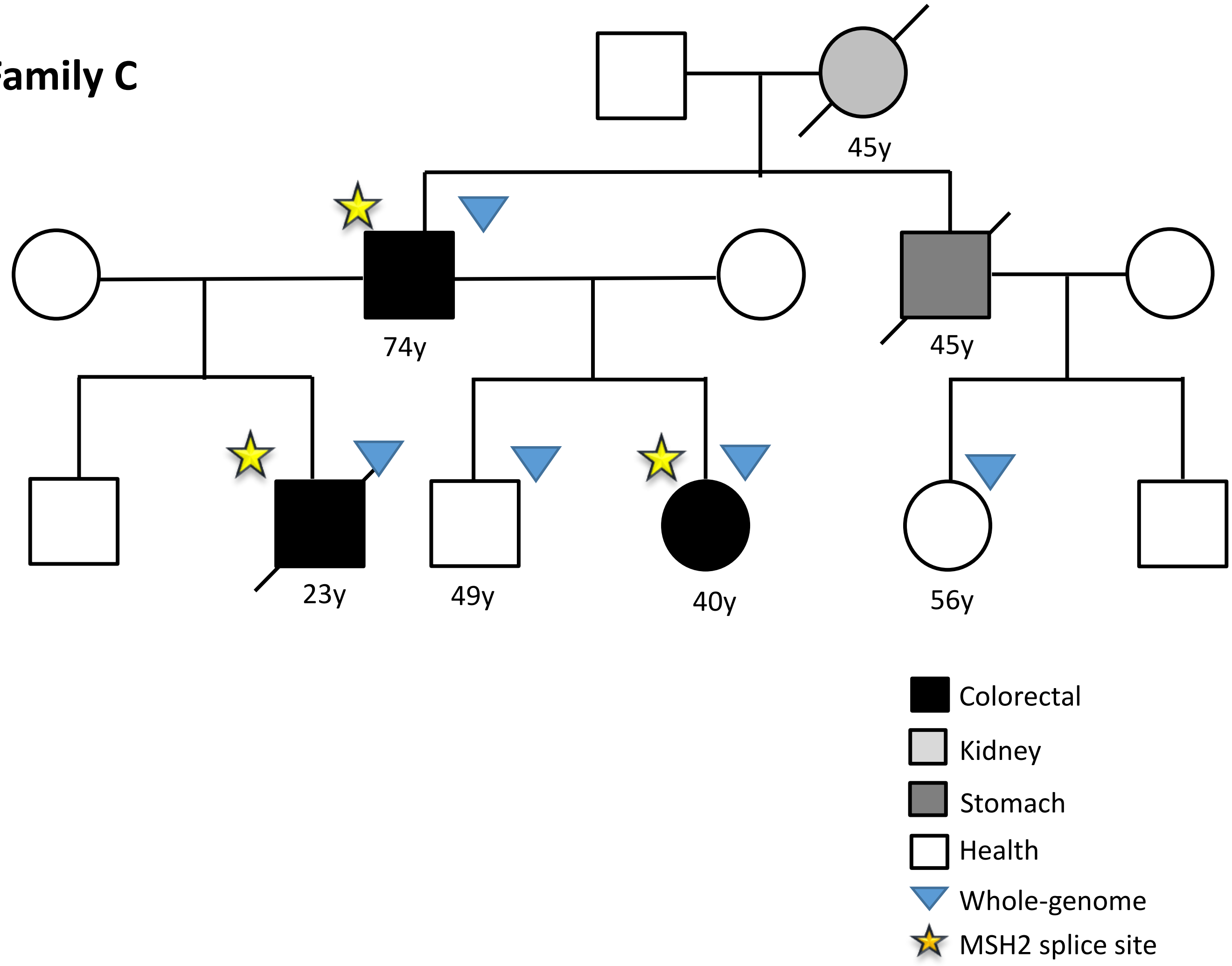
45y
74y
45y
23y
49y
40y
56y
Colorectal
Kidney
Stomach
Health
Whole-genome
MSH2 splice site

Additional file: Fig. 2 Microsatellite instability (MSI) analysis of tumor samples of two family members from Family A and one tumor sample from Family C. For each family, individuals with tumor samples analyzed are indicated by an arrow and the MSI plots are shown for the corresponding germline and tumor samples.

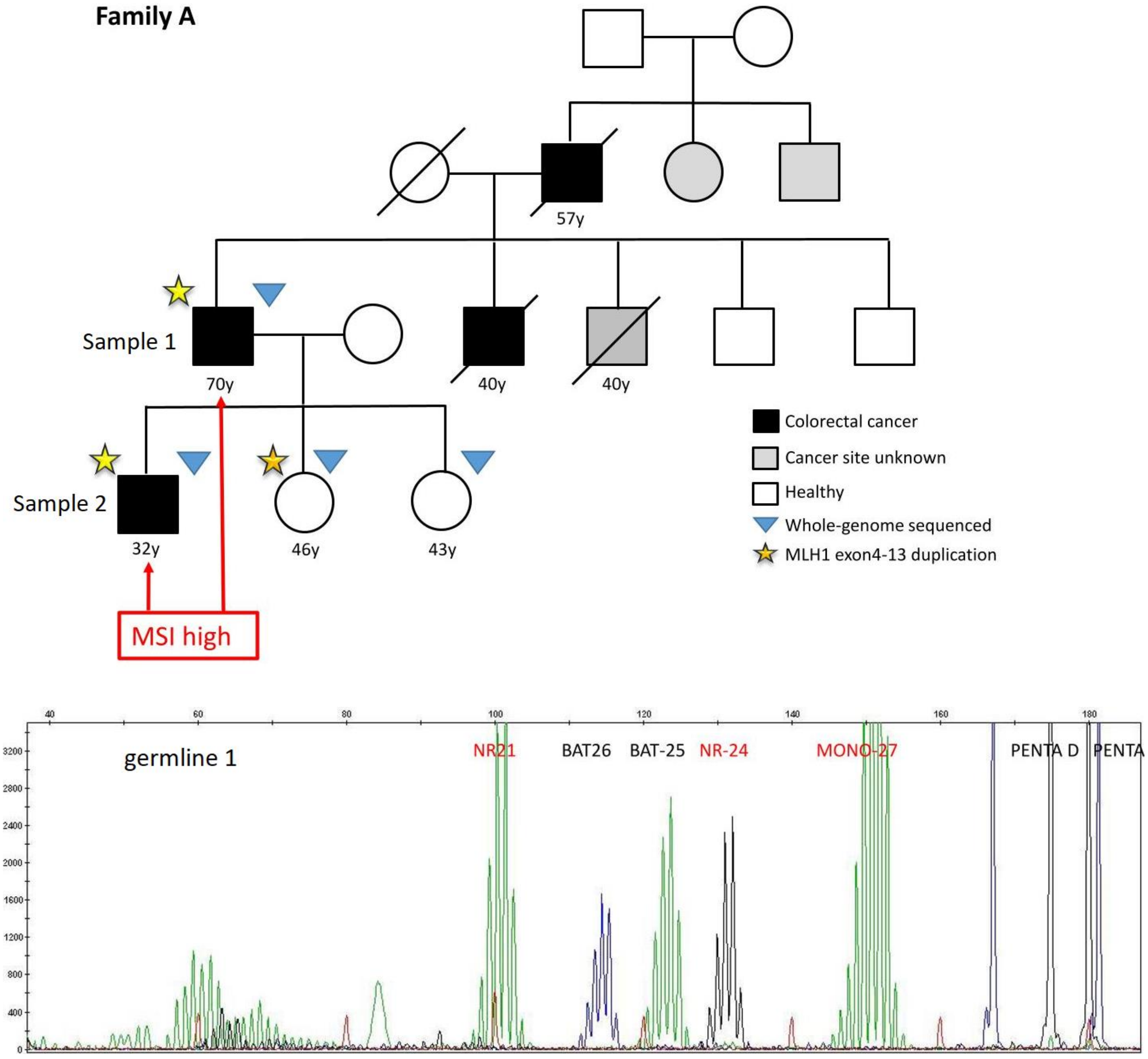

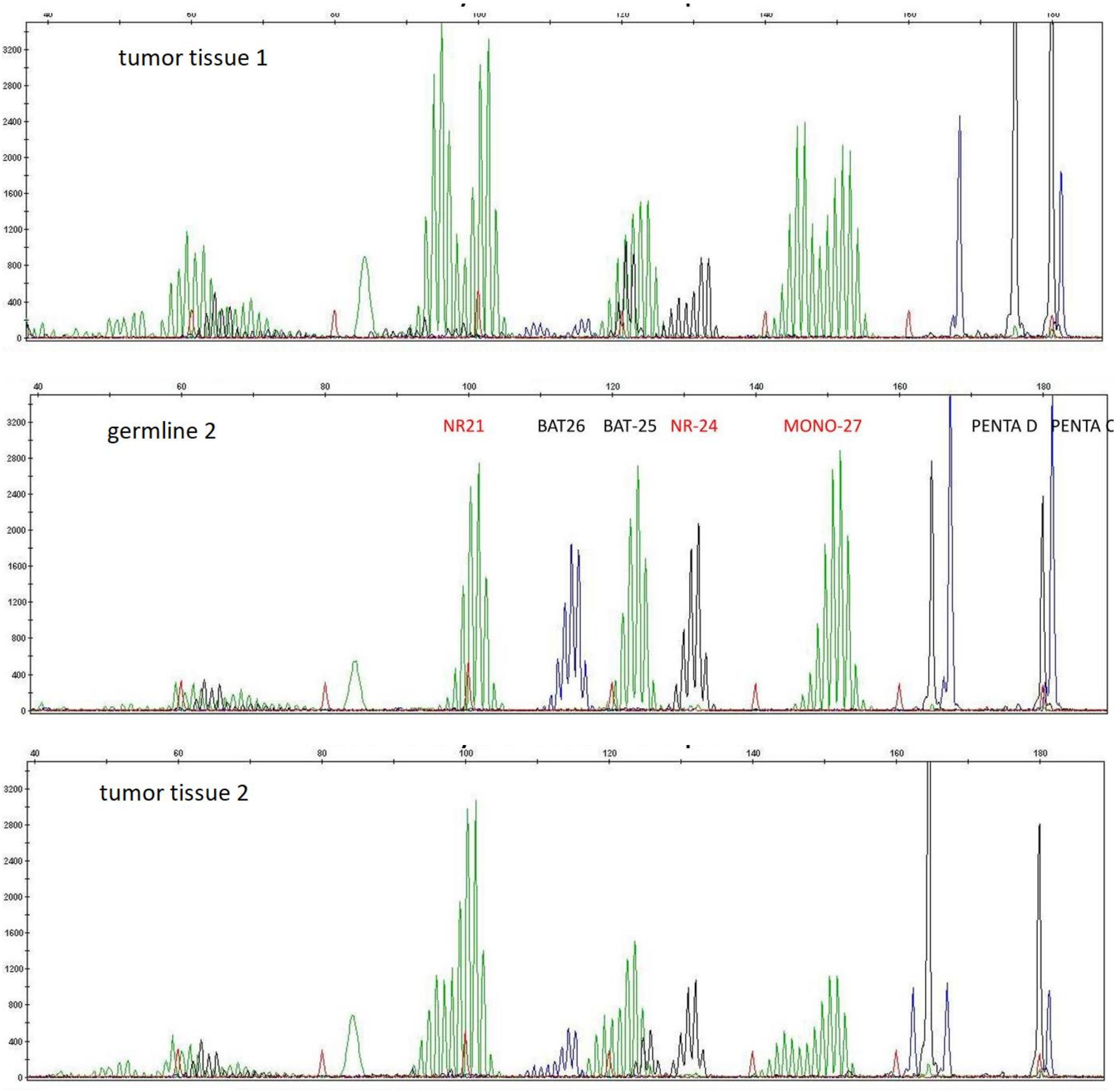
tumor tissue 1
germline 2
NR21
BAT26
BAT-25
NR-24
MONO-27
PENTA D
PENTA C
tumor tissue 2

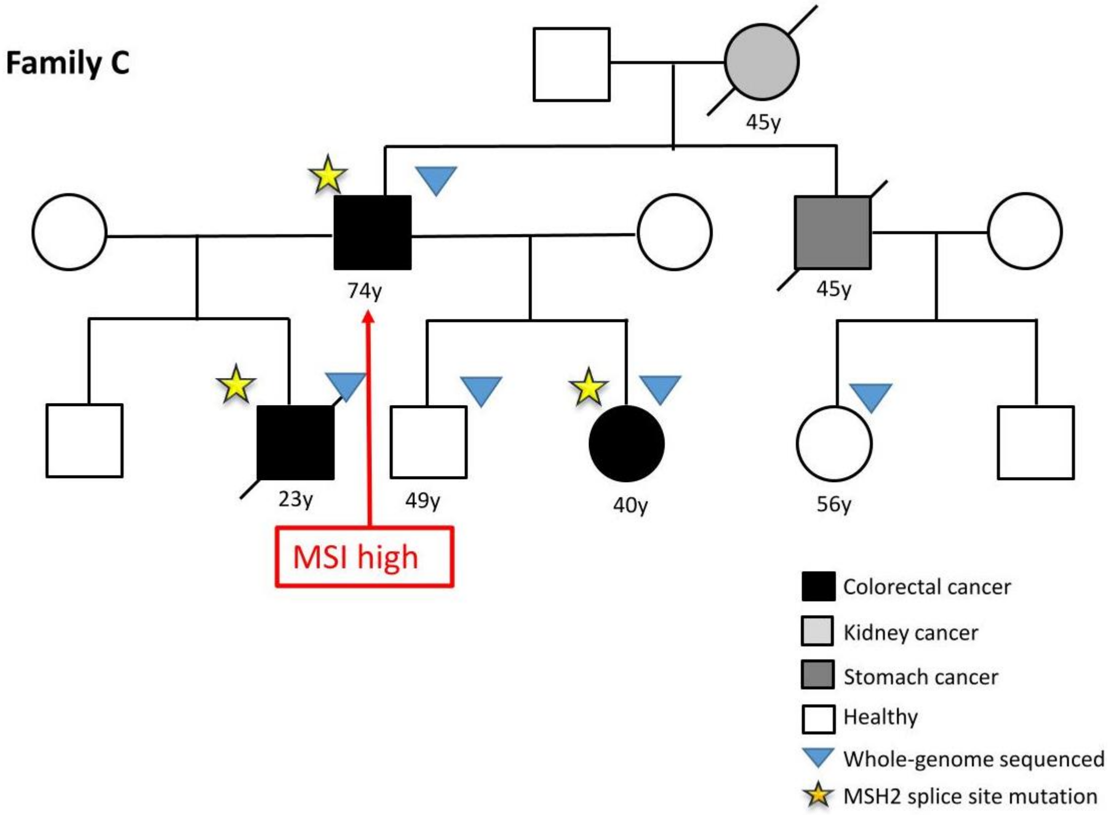
Family C
45y
74y
45y
23y
49y
40y
56y
MSI high
Colorectal cancer
Kidney cancer
Stomach cancer
Healthy
Whole-genome sequenced
MSH2 splice site mutation

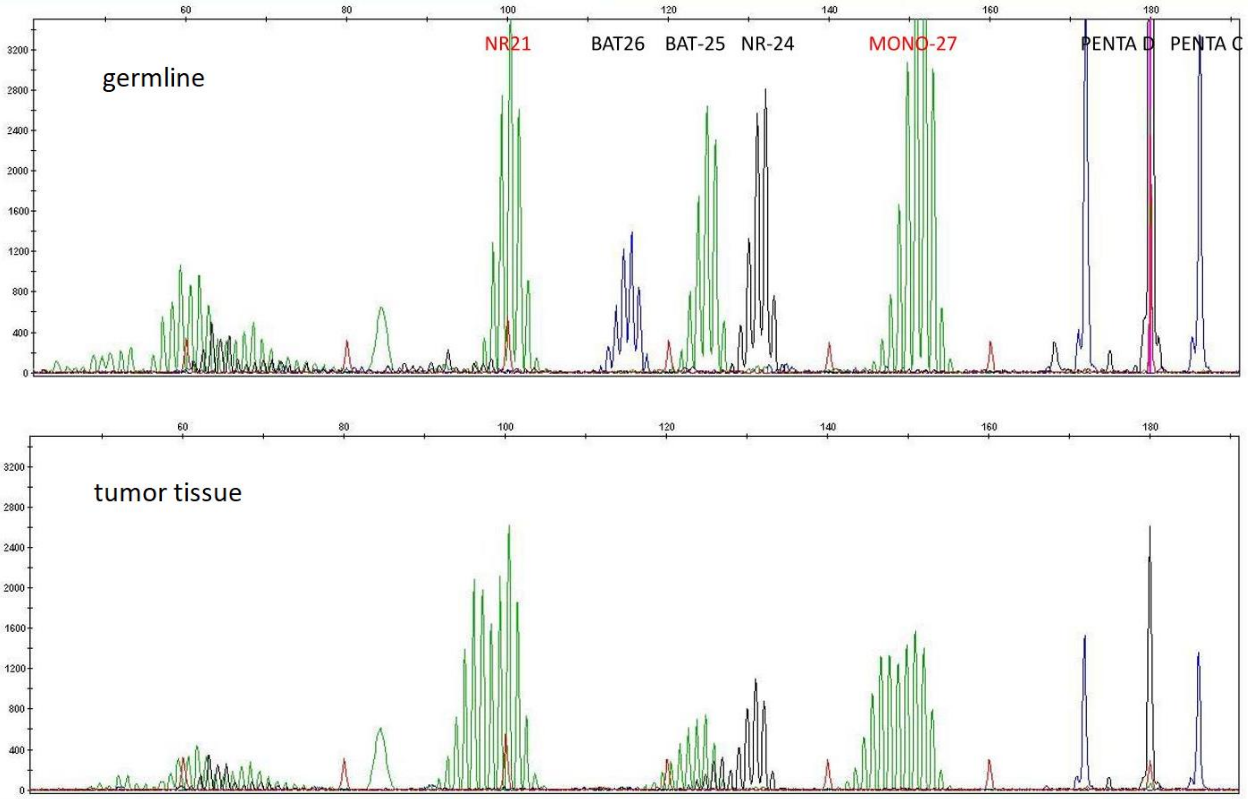
germline
NR21
BAT26
BAT-25
NR-24
MONO-27
PENTA D
PENTA C
tumor tissue
